# Effects of Break Crops on Yield and Grain Protein Concentration of Barley in a Boreal Climate

**DOI:** 10.1371/journal.pone.0130765

**Published:** 2015-06-15

**Authors:** Ling Zou, Markku Yli-Halla, Frederick L. Stoddard, Pirjo S. A. Mäkelä

**Affiliations:** 1 Department of Agricultural Sciences, University of Helsinki, Helsinki, Finland; 2 Department of Food and Environmental Sciences, University of Helsinki, Helsinki, Finland; Institute for Sustainable Plant Protection, C.N.R., ITALY

## Abstract

Rotation with dicotyledonous crops to break cereal monoculture has proven to be beneficial to successive cereals. In two fields where the soil had been subjected to prolonged, continuous cereal production, two 3-year rotation trials were established. In the first year, faba bean, turnip rape and barley were grown, as first crops, in large blocks and their residues tilled into the soil after harvest. In the following year, barley, buckwheat, caraway, faba bean, hemp and white lupin were sown, as second crops, in each block and incorporated either at flowering stage (except barley) or after harvest. In the third year, barley was grown in all plots and its yield and grain protein concentration were determined. Mineral N in the plough layer was determined two months after incorporation of crops and again before sowing barley in the following year. The effect of faba bean and turnip rape on improving barley yields and grain protein concentration was still detectable two years after they were grown. The yield response of barley was not sensitive to the growth stage of second crops when they were incorporated, but was to different second crops, showing clear benefits averaging 6-7% after white lupin, faba bean and hemp but no benefit from caraway or buckwheat. The effect of increased N in the plough layer derived from rotation crops on barley yields was minor. Incorporation of plants at flowering stage slightly increased third-year barley grain protein concentration but posed a great potential for N loss compared with incorporation of crop residues after harvest, showing the value of either delayed incorporation or using catch crops.

## Introduction

Cereals are important sources of carbohydrate and protein for both humans and animals, but continuous cereal production has negative impacts on soil fertility and health. For example, 20 to 70 years of continuous cereal production in six regions of Australia was associated with, on average, a 70% loss of total soil N from sandy soil, 38% from silty soil and 18% from clay soil [1]. In soils under continuous cereal production in the UK, organic C concentration decreased from around 50 Mg/ha in 1880 to 20 Mg/ha in 1980 [2]. In addition, cereal-specific pathogen inocula increase with long-term continuous cereal production [3].

In Finland, 46% of the arable land is used for continuous production of cereals, with barley (*Hordeum vulgare* L.) being the most produced cereal crop [4]. Introducing dicotyledonous crops as breaks into cereal-based cropping sequences brings benefits such as decreased pathogen pressure and improved soil fertility, with different break crops having different effects. *Brassica* crops such as turnip rape [*Brassica rapa* L. ssp. *oleifera* (DC.) Metzg.] offer biofumigation potential [5], but generally do not support mycorrhizal fungi. Legumes such as faba bean (*Vicia faba* L.) and white lupin (*Lupinus albus* L.) support the growth of beneficial soil microbes [6], in addition to providing biological nitrogen fixation. White lupin, buckwheat (*Fagopyrum esculentum* L.), caraway (*Carum carvi* L.), and hemp (*Cannabis sativa* L.) all contain secondary compounds that may inhibit the growth of weeds or subsequent crops [7–10], because allelochemicals, most of which are secondary compounds, are involved in competition between different plant species [11]. All of these dicotyledonous crops have strong tap roots that can improve soil permeability [12]. Incorporation of break-crop plant material as a green manure is done at the expense of harvestable grain or seed yield, but maximizes the input to the soil of both slowly degraded organic N and secondary chemicals that may affect soil microbiological community structure. Many secondary metabolites of plants are inhibitory to a wide range of pathogenic fungi such as *Fusarium culmorum* and *Rhizoctonia solani* [13]. The concentrations of these metabolites are generally highest in plants at the flowering stage [14], and the incorporation of green manure at this stage offers the further advantage that it is unlikely to interfere with the sowing time of the next crop.

For these reasons, we set out to investigate the effects of a range of rotation crops, managed for either grain yield or green manure, on the yield and quality of barley, with continuous barley as a control. Mineral N concentration in the plough layer in soil was determined between incorporation of plant materials and sowing barley in the following spring, in order to evaluate the potential for loss of N mineralized from plant residues. The experiment was repeated with a one-year lag in order to test the potential for generalizing the results within a manageable time-frame.

## Materials and Methods

### Experimental site

Two rotation experiments were conducted in 2010–2012 and 2011–2013 on two sites at the Viikki Experimental Farm (60°22' N, 25°03' E, 8 m amsl), University of Helsinki, Finland, at the southern edge of the Boreal climatic zone. Both sites had primarily been used for cereal production and were classified as Vertic Stagnosols [15], with a topsoil of silty clay loam comprised of 31–33% clay, 63% silt and 4–5% sand. The pH(H_2_O) of the plough layer of the natively acidic soils had been raised to 6.4 with repeated liming for 50 years. Soil total C and N concentrations were analyzed with the Dumas dry combustion method in a VarioMAX CN analyzer (Elementar Analysensysteme GmbH, Hanau, Germany). Carbonate content in these soils is too low to be detectable, and the C and N are mainly in organic forms. C concentrations were 38.5 and 74.6 g/kg, and N concentrations were 3.4 and 6.6 g/kg in site I (2010–2012) and II (2011–2013), respectively, giving a C/N ratio of about 11.3.

### Experimental set-up, sampling and analysis

**Fig 1:**
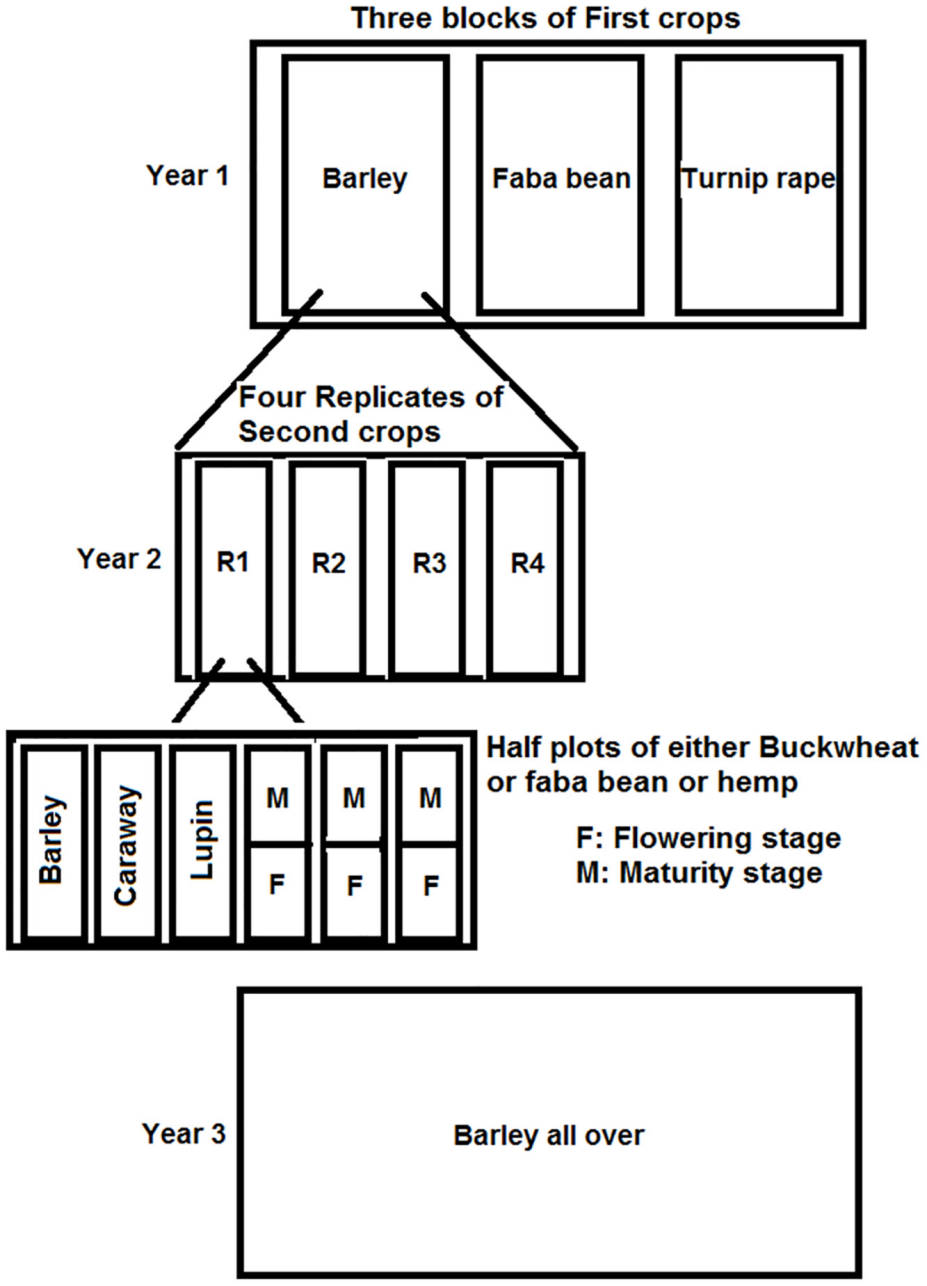
Field experimental setup. Barley, faba bean and turnip rape, as first crops, were grown in one block in the first year. In the second year, second crops were sown in randomized complete block designs with four replicates in each block of First crops. The whole plots of caraway and white lupin were incorporated into the soil at flowering stage, and the residues of the whole plots of barley were incorporated after harvest. Half plots of buckwheat, faba bean and hemp were incorporated into the soil at flowering stage and the residues of the other half were incorporated after harvest. In the third year, all the plots were sown with barley. The first year of site II coincided with the second year of site I.

Throughout the experiment in Table 1, the synthetic fertilizer, banded to the seed-bed at the time of sowing, was N-P-K 23-3-5 (Cemagro Oy, Lohja, Finland) for the non-legume crops and N-P-K 16-7-13 for the legumes. Both faba bean and white lupin were inoculated with the appropriate commercial strains of rhizobium (Elomestari Oy, Kukkola, Finland) before sowing. In the first year at both sites, three blocks (40 m × 25 m) of turnip rape (cv ‘Wildcat’), faba bean (cv ‘Kontu’) and barley (cv ‘Vilde’) were sown as shown in Fig 1. These "first crops" were harvested in late August, after which the plots were ploughed.

**Table 1:** Crops, fertilizer use (N kg/ha) and sowing density (viable seeds/m^2^) in the experiments (2010–2012, 2011–2013).

Crops	Fertilizer N (kg/ha)	Sowing density (viable seeds/m^2^)
First year		
Barley	90	400
Faba bean	20	80
Turnip rape	90	150
Second year		
Barley	90	400
Buckwheat	60	200
Caraway	50	50
Faba bean	20	80
Hemp	60	170
White lupin	20	90
Third year		
Barley	60	400

In the second year, the block was divided into the four replicates of a randomized complete block design, with the treatments being the "second" crops, namely caraway cv 'Sylvia', white lupin cv 'Vesna', oilseed hemp cv 'Finola', a landrace of buckwheat, and the same barley and faba bean cultivars as before as shown in Fig 1. Each plot was 6 m × 2.5 m and seeding densities and fertilizer rates are shown in Table 1. Sowing was in late May of each year.

At full flowering stage (BBCH growth stage (GS) 65 [16]), the entire plots of caraway and white lupin, and half of each plot of buckwheat, faba bean and hemp were incorporated into the soil using a rotary power harrow in the middle of July. The whole plots of caraway and white lupin were used because they were considered very unlikely to reach maturity in this climate. The residues of the other half-plots of buckwheat, faba bean and hemp were incorporated into the soil at maturity (GS 89), after the seeds were harvested in late August. The barley plots were harvested normally and their stubble and straw were incorporated into the soil. Two months after the incorporation of plant materials, topsoil samples (0–30 cm) were taken for mineral N analysis (N.2).

In the third year, samples of topsoil (0–30 cm) were taken from four random points within each block, using an auger in early May before seeding. Samples were stored at -20°C until they were analysed at the Finnish Environmental Services (Suomen Ympäristöpalvelu Oy, Oulu, Finland) for ammonium and nitrate N (N.BS) using a FIAstar 5000 analyzer (Foss Tecator AB, Höganäs, Sweden). At the end of May, all the plots were sown with barley and fertilized with 60 kg/ha of N. Normal N fertilization of barley in this region is 100 kg/ha, so the amount used was chosen to allow detection of response to mineral N from other sources. In late August, the barley was harvested from the middle rows of all plots. The yield (kg/ha) was determined from 7.5 m^2^ (6 m × 1.25 m) of each plot where the second crop was treated as a whole plot (caraway, white lupin and barley), and from 3.75 m^2^ (3 m × 1.25 m) of each plot where the second crop was treated as a half-plot (buckwheat, faba bean and oilseed hemp, incorporated at either the flowering or after harvest). Grain protein concentration (GPC, %) was determined by near infra-red reflectance analysis (DA 7200, Perten Instruments AB, Segeltorp, Sweden). Weather data of 2010–2013 were obtained from the Finnish Meteorological Institute (http://en.ilmatieteenlaitos.fi/) station at Kaisaniemi, 9 km from the sites, including daily temperature (°C) and monthly rainfall (mm).

### Data analysis

The difference (N.D) between N.2 and N.BS was calculated as a measure of the potential for N loss. Data on barley yield, GPC, N.2, N.BS, and N.D from across the two sites were subjected to a full factorial analysis of variance using R [17], with site, first crop, second crop, and growth stage of second crop incorporation and their interactions as fixed factors, and replicate as a random factor. Replicate number was nested within each first-crop block. The normality of data of models was checked by quantile-quantile (Q-Q) plots. The full-factorial models were then simplified stepwise by dropping first the random and mixed effects and then the fixed effects, starting with the highest order interactions. At each step, the simplified model was compared with the previous one, to determine whether the change was significant (*P* < 0.05). Means of the retained fixed effects were compared with the LSD test (*P* < 0.05) in the agricolae package [18]. For analysis of covariance (ANCOVA) and regression analysis of barley yield, N.BS was added as a covariate to the simplified models or as an explanatory variable to models with only an intercept, for the sites separately. Total precipitation as rainfall (mm), the number of frost days ≤0°C, and the average daily temperature (°C) were calculated from May to October and November to April.

## Results

Data of dependent variables were normally distributed. The block structure of the experiment (i.e., the replicate × site × first crop interaction) was significant only in the analysis of N.D as shown in Table 2 (mean squares were shown in S5 Table), easing the analysis and interpretation of the fixed effects on yield, GPC, N.2 and N.BS.

**Table 2:** Significance levels of terms included in the final models used in the analysis of variance of yield and grain protein concentration (GPC) of barley along with soil mineral nitrogen concentrations two months after incorporation of second crops (N.2), before sowing the final barley crop (N.BS), and difference between incorporation and sowing (N.D).

Source of variation	DF	Yield	GPC	N.2	N.BS	N.D
Site	1	***		***	***	***
First crop	2	***	***	***	***	*
Second crop	5	**	***	***	***	***
Stage of incorporation	1		***	***	*	***
Site × First crop	2			***	**	***
Site × Second crop	5			***	***	
First crop × Second crop	10	*	*			
Site × Stage of incorporation	1			***	***	***
First crop × Stage of incorporation	2			*		*
Second crop × Stage of incorporation	2		***	**		
Site × First crop × Second crop	10				**	
Site × First crop × Stage of incorporation	2			*		**
Site × Second crop × Stage of incorporation	2					
First crop × Second crop × Stage of incorporation	4	*				
Site × First crop × Second crop × Stage of incorporation	4				*	
Replicate	3					
Residual	159					

*, **, ***: P < 0.05, 0.01, 0.001, respectively.

### Effect of sites, first crops and their residues on soil mineral N and barley yield and grain protein

Barley yield, N.2 and N.D were significantly (*P* < 0.05, Table 2) higher at site I (2011–2012) than at site II (2012–2013) as shown in Table 3, but N.BS was significantly higher at site II. In both sites, N.2, N.BS and N.D were lowest in the barley block as shown in Table 3, but its highest value was in different treatments in the two sites, leading to a significant site × first crop interaction as shown in Table 2 and S1 Table. Yields and GPC of barley were lower in the barley block than blocks of faba bean and turnip rape at both sites as shown in Table 3.

**Table 3:** Barley yields, GPC, mineral nitrogen concentration two months after incorporation of plant materials (N.2), before sowing the barley crop (N.BS), and difference (N.D) between N.2 and N.BS, as affected by site, first crop, second crop, and stage of incorporation of second-crop residues. Data show main effect means.

	Yield (kg/ha)		GPC (%)		N.2 (kg/ha)		N.BS (kg/ha)		N.D (kg/ha)	
Site										
I	5628	a			46.9	a	23.4	b	23.4	a
II	4616	b			35.5	b	29.2	a	6.3	b
First crop										
Barley	4906	b	8.9	c	35.8	b	24.3	b	11.5	b
Turnip rape	5203	a	10.0	a	43.9	a	27.7	a	16.2	a
Faba bean	5258	a	9.6	b	43.8	a	26.8	a	17.0	a
Second crop										
Barley	4914	c	9.4	bc	37.3	cd	22.3	c	15.0	b
Buckwheat	5000	bc	9.4	bc	36.7	cd	25.8	b	10.9	b
Caraway	5115	abc	9.5	bc	45.3	b	26.7	ab	18.6	b
Faba bean	5256	a	9.8	a	40.8	bc	29.2	a	11.6	b
Hemp	5190	a	9.3	c	33.9	d	25.5	b	8.4	b
White lupin	5179	ab	9.6	b	65.7	a	26.7	ab	39.0	a
Stage of incorporation										
Flowering			9.6	a	53.4	a			27.2	a
After harvest			9.4	b	25.9	b			-0.5	b

Within a column, means followed by the same letter are not significantly different (*P* < 0.05) by the LSD test.

### Effects of first crops, second crops, and the timing of their incorporation and their interactions on soil mineral N, barley yield and quality

Although barley yields were not significantly affected by the timing when the second crops were incorporated, N.2 and N.D were higher when incorporation took place at flowering stage than after harvest as shown in Table 3, indicating the higher potential for loss of mineral N from the plough layer in this circumstance. Nevertheless, the higher figure for GPC after incorporation at flowering suggests that at least some of the organic N became available at grain-filling time.

**Fig 2:**
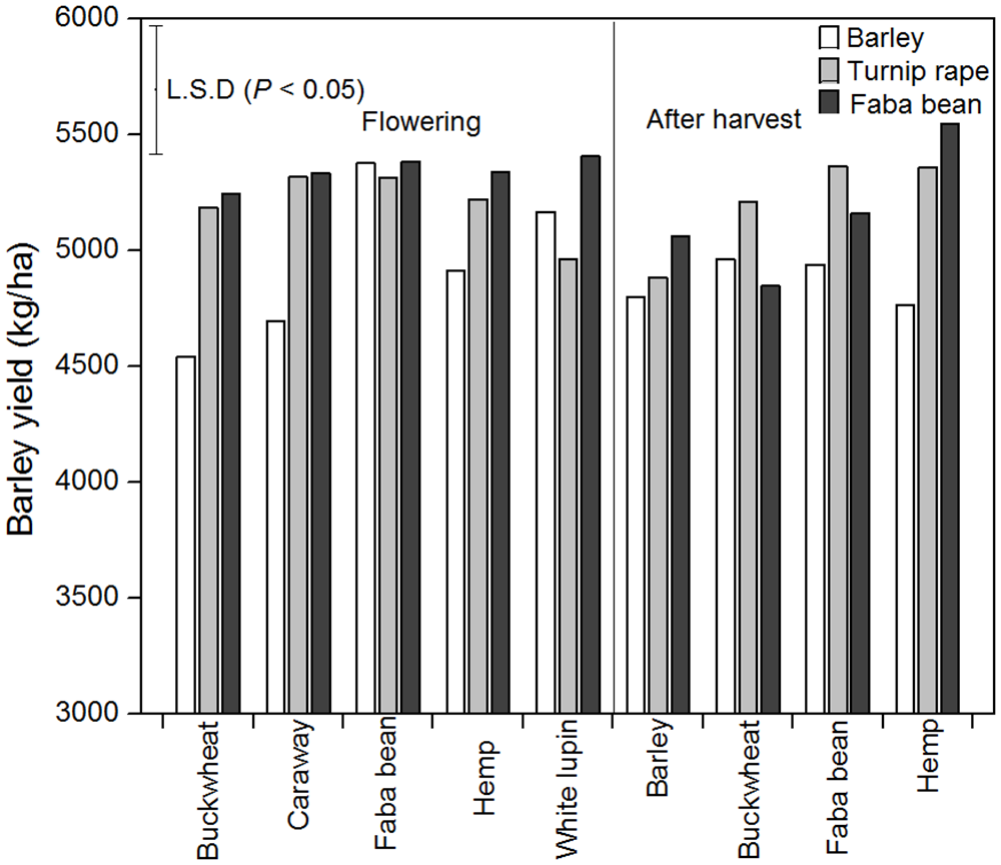
Yields of barley as affected by first crop, second crop and stage of incorporation of the second crop.

Barley yield was significantly affected by the first crop × second crop and first crop × second crop × stage of incorporation interactions as shown in Table 2, S3 Table and Fig 2. The four lowest barley yields were in the block of barley as first crop as shown in Fig 2, with the second crops being buckwheat (flowering stage), caraway, hemp (after harvest) and barley. Barley yields were generally highest after a two-year break, with the top three yields following a first crop of faba bean and a second crop of hemp (after harvest), white lupin (flowering) or faba bean (flowering) as shown in Fig 2.

GPC was significantly affected by the first crop × second crop and second crop × stage of incorporation interactions as shown in Tables 2, 4, and S3 Table. GPC was higher after faba bean incorporated at flowering than in any of the other second crop × incorporation treatments as shown in Table 4 and was higher when faba bean was the second crop after any first crop as shown in S3 Table. The first crop × second crop interaction was particularly evident for the white lupin treatment, that gave a high GPC after turnip rape but a very low one after barley as shown in S3 Table.

**Table 4:** Grain protein content of barley and soil nitrogen content two months after incorporating the second crop, according to the second crop and its stage of incorporation.

Stage of incorporation	Second crop	GPC (%)		N.2 (kg/ha)	
Flower	Buckwheat	9.4	b	49.5	b
	Caraway	9.5	b	45.3	bc
	Faba bean	10.2	a	60.6	a
	Hemp	9.3	b	46.0	bc
	White lupin	9.6	b	65.7	a
After harvest	Barley	9.4	b	37.3	c
	Buckwheat	9.4	b	23.9	d
	Faba bean	9.5	b	20.9	d
	Hemp	9.3	b	21.7	d

Data show means across first crops and sites.

Within a column, means followed by the same letter are not significantly different (P < 0.05) by the LSD test.

N.2, N.D and N.BS were all affected by the site × first crop as shown in S1 Table and site × second crop as shown in S2 Table interactions along with several other two-way and three-way interactions as shown in Tables 2, 4 and S4 Table. N.2 and N.D were both high after white lupin as shown in Table 5, especially at the second site as shown in S2 Table, and N.2 was also high after faba bean incorporated at the flowering stage as shown in Table 4. N.2 and N.D were remarkably low at site 2 in the materials incorporated after harvest, as shown in S4 Table. N.BS was low at site 1 after barley and faba bean as shown in S1 Table. It was low after barley at both sites, but after white lupin, it was high at site 2 and low at site 1 as shown in S2 Table.

**Table 5:** Soil nitrogen content two months after incorporation of crop residues (N.2), soil nitrogen content before sowing barley (N.BS), and difference between N.2 and N.BS (N.D).

First crop	Growth stage	Second crop	Site I 2012						Site II 2013					
			N.2	(kg/ha)	N.BS	(kg/ha)	N.D	(kg/ha)	N.2	(kg/ha)	N.BS	(kg/ha)	N.D	(kg/ha)
Barley	Flowering	Buckwheat	39.8	gh	19.9	gh	19.9	efgh	44.6	bcd	24.8	ef	19.8	abcde
		Caraway	34.7	gh	22.8	cdefg	11.9	ghij	39.2	bcde	25.8	def	13.4	bcdef
		Faba bean	37.2	gh	27.0	bc	10.2	ghij	58.9	ab	25.0	ef	33.9	ab
		Hemp	33.5	gh	18.4	gh	15.2	fghij	38.4	cde	25.8	def	12.6	abcde
		White lupin	46.1	efg	15.4	h	30.8	bcde	74.5	a	33.2	abcde	41.3	abc
	After harvest	Barley	32.6	h	18.3	gh	14.3	fghij	41.8	bcde	23.7	f	18.2	bcde
		Buckwheat	32.8	h	18.3	gh	14.5	fghij	11.5	fg	31.9	abcdef	-20.4	hi
		Faba bean	30.5	h	20.7	fg	9.8	ghij	9.6	g	38.3	ab	-28.7	i
		Hemp	29.7	h	19.0	gh	10.8	ghij	10.2	fg	29.8	bcdef	-19.6	ghi
Turnip rape	Flowering	Buckwheat	71.5	ab	26.1	bcd	45.4	ab	44.3	bcde	27.4	cdef	16.9	bcde
		Caraway	64.4	abcd	22.5	cdefg	41.9	abc	36.8	de	36.3	abc	0.6	efg
		Faba bean	66.4	abcd	38.3	a	28.1	cdef	58.2	abc	30.7	bcdef	27.5	abc
		Hemp	62.1	bcd	24.8	bcdef	37.3	abcd	40.2	bcde	25.5	ef	14.7	bcdef
		White lupin	68.0	abc	25.1	bcdef	43.0	ab	67.0	a	27.5	cdef	39.6	abcd
	After harvest	Barley	41.5	fgh	18.6	gh	23.0	defg	30.3	def	24.6	ef	5.7	def
		Buckwheat	38.7	gh	27.8	b	11.0	ghij	13.0	fg	29.1	cdef	-16.1	ghi
		Faba bean	32.5	h	27.1	bc	5.4	ij	10.8	fg	28.7	cdef	-17.9	ghi
		Hemp	36.6	gh	27.0	bc	9.7	hij	8.8	g	32.0	abcdef	-23.2	hi
Faba bean	Flowering	Buckwheat	56.4	cde	21.4	efg	35.1	abcd	40.3	bcde	24.8	ef	15.5	bcdef
		Caraway	56.2	cde	22.0	defg	34.2	bcd	40.6	bcde	31.0	bcdef	9.6	cdef
		Faba bean	76.0	a	27.9	b	48.1	a	67.2	a	28.8	cdef	38.4	a
		Hemp	60.5	bcd	25.5	bcde	35.1	abcd	41.7	bcde	23.3	f	18.4	abcde
		White lupin	60.7	bcd	19.5	gh	41.2	abc	77.9	a	40.0	a	38.0	bcde
	After harvest	Barley	53.8	def	21.4	defg	32.4	bcde	23.9	efg	27.4	cdef	-3.6	fgh
		Buckwheat	36.5	gh	28.5	b	8.0	hij	11.1	fg	29.7	bcdef	-18.6	ghi
		Faba bean	30.9	h	28.4	b	2.6	j	11.5	fg	29.4	bcdef	-17.9	ghi
		Hemp	37.3	gh	20.6	fg	16.7	fghi	7.7	g	34.6	abcd	-27.0	i
		LSD (0.05)	13.3		4.7		13.8		20.5		8.9		22.9	

Data show means of each second crop at each stage of incorporation at each site (n = 4, with LSD (Least significant difference, *P* = 0.05).

Within a column, means followed by the same letter are not significantly different (*P* < 0.05), negative numbers of N.D indicate gain of mineral N.

**Fig 3:**
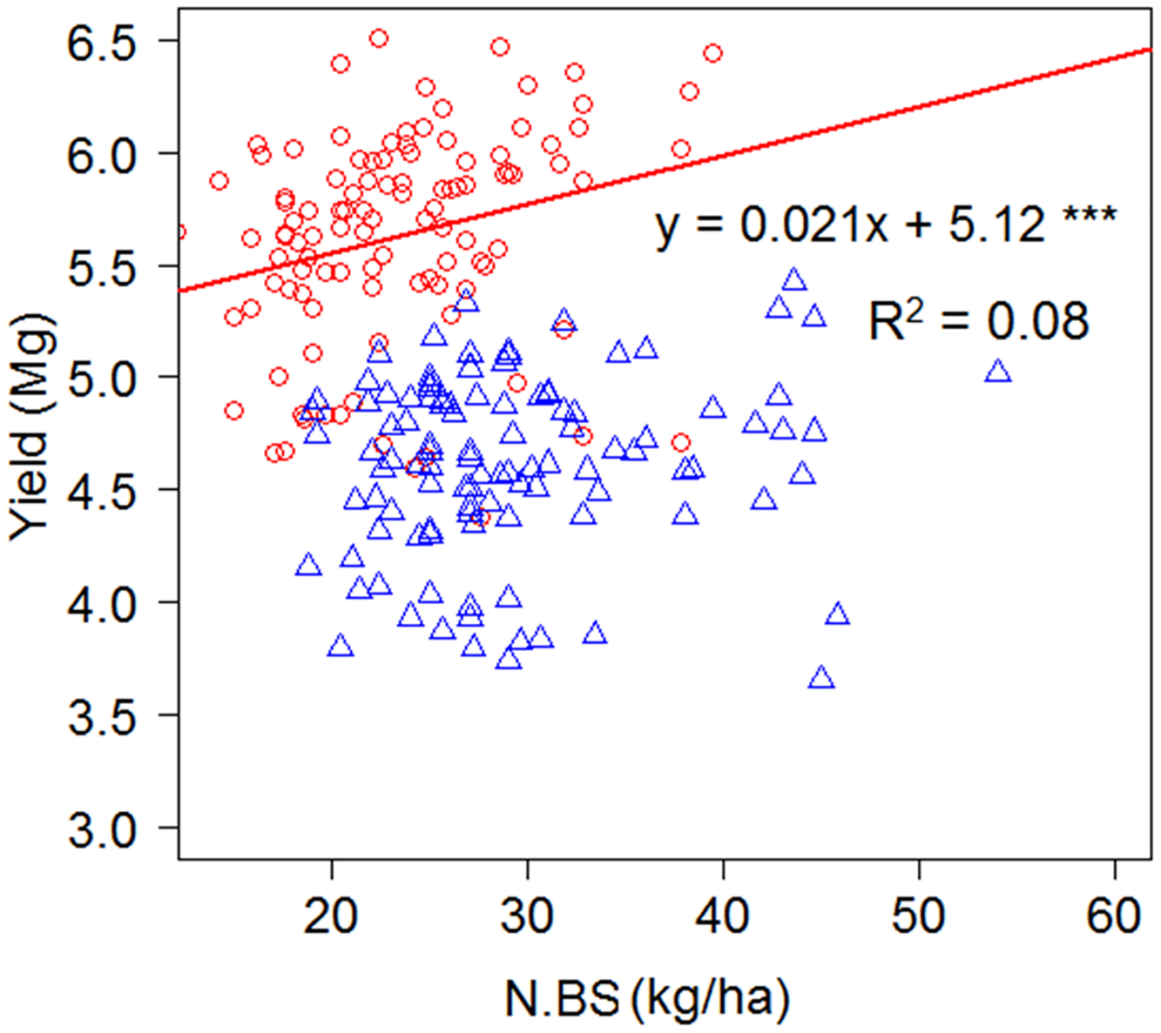
Response of barley yields to mineral N in the plough layer (N.BS) before sowing third-year barley. The correlation was significant at site I, with the S.E. of the slope being 0.008 and that of the intercept 0.19.

Barley yield showed a weak correlation with N.BS in Site I as shown in Fig 3, and was, in general, higher at Site I than at Site II. N.BS added as a covariate to the simplified models did not cause any significant change of variation explained by the models.

### Weather conditions between harvest and sowing

The average daily temperature show that May—October 2012 was cooler than the other two growing seasons, whereas winter 2011–2012 was warmer than the other two in Table 6. The single days of frost in the two growing seasons were during October, after harvest. Summer of 2010 and winter 2010–2011 were much dryer than the subsequent years.

**Table 6:** Average daily temperature (°C), number of frost days, and cumulative precipitation as rainfall (mm) during and between the growing seasons.

	Average daily temperature (°C)	Frost days (number)	Precipitation (mm)
2010 (May–Oct)	13.9	1	277
2010–2011 (Nov–Apr)	-3.8	128	266
2011 (May–Oct)	14.4	0	373
2011–2012 (Nov–Apr)	-0.6	80	424
2012 (May–Oct)	12.7	1	518
2012–2013 (Nov–Apr)	-2.8	125	299

## Discussion

The effect of faba bean as first crop on increasing barley yield, and its grain protein concentration, were both still detectable after two years. Faba bean, hemp and white lupin as second crops were all associated with higher third-crop barley yield. In contrast, the effect of soil mineral N in the plough layer before sowing was minor. The ranges of mineral N in the plough layer of the present study were comparable with other studies conducted in similar environments [19–21]. When plants were incorporated at flowering stage as green manure, there was a substantial decrease in the mineral N concentration between two months after the incorporation and the next spring, posing a great potential for N loss from the plough layer at this time. Green manure incorporation, in general, increased third-year barley GPC, but did not result in more mineral N before sowing barley or higher third-year barley yields than residue incorporation after harvest. Mineral N concentration before sowing barley in the plough layer was apparently affected by weather factors such as the number of days with temperature below 0°C, average daily temperature and total winter rainfall.

### The benefits of faba bean and turnip rape on increasing yields and GPC of barley were still detectable two years later

Faba bean and turnip rape, as first crops, improved yields and grain protein concentration (GPC) of successive barley. Rapeseed has been reported to enhance the growth and grain yield of subsequent wheat [22]. Faba bean increased soil mineral N and enhanced N uptake by the subsequent wheat in Germany [23]. The positive effect of turnip rape on GPC was greater than that of faba bean.

### Faba bean, hemp and white lupin as second crops resulted in higher third-year barley yields and grain protein concentration

In general, barley yields after a two-year break were higher than after a one-year break, indicating that the beneficial effect of rotation crops could be cumulative. Faba bean, hemp and white lupin as second crops were all associated with higher third-crop barley yields, whereas buckwheat and caraway resulted in lower barley yields, confirming that the beneficial effects of rotation crops differ. Although we have previously found [24] that buckwheat can inhibit weed growth because of its large leaf area and root exudates containing allelochemicals, it is not evident if the residual effect of these exudates or other secondary chemicals caused the low barley yield in this study.

Increased N availability in the plough layer is one of the contributors of break crops to barley yields, as it has been shown to account for increased yields or promoted growth of many following crops [25–30]. However, its effect can be overestimated, as shown by the lack of significant effect of N.BS in the ANCOVA. Moreover, the regression model using N.BS to predict barley yield was not significant in site II and was only weak in site I. The significant positive correlation between barley yield and N.BS in site I suggested that the application of 60 kg/ha of N fertilizer was appropriate to allow detection of additional available N. In a meta-analysis of Finnish data from 1940–2004, that barley yield response to N fertilization and N derived from soil organic matter varied between years, and that the variation in yield was largely due to unidentified factors [31].

For prospective studies, it would be useful to investigate other components of the break crop effects of these species in this climate, such as reduced pathogen populations, improved soil structure, and other nutritional aspects such as the solubilization of P by carboxylates released by legume roots [32, 33].

### Incorporation of green manure improved barley grain protein concentration but did not increase N.BS and barley yields

Plants incorporated at the flowering stage contained more nutrients than those incorporated after harvest, which may account for the increase of barley GPC, but third-year barley yield did not show differences due to incorporation stage. Therefore, incorporation at flowering stage at the expense of harvest may not be recommendable. Moreover, green manure incorporation can pose a great potential for N leaching, since mineral N concentration before sowing third-year barley was about half of the amount two months after incorporation. This decrease of mineral N concentration could be due to immobilization, gaseous loss, and particularly to leaching. The average temperature between harvesting and sowing in this region is about -0.5°C at 20 cm depth [34], and this is considered to preclude significant microbiological activity that could lead to immobilization [35]. N leaching in spring during snow-melt, in contrast, is a very well established source of N losses [36]. Furthermore, mineralization is predominant over immobilization in early spring in the boreal climate of this area, by about 10 kg/ha [37], adding to the risk of leaching losses.

Since the soil temperature averaged around 8°C from July to late October [34], N from organic matter would have continued to be mineralized and hence more subject to loss. This might also explain how N concentration fell more when plants were incorporated at flowering stage. Incorporation of plants after harvest took place in late August, so the organic N of the residues would have been less subject to mineralization. Moreover, the lack of plant cover that holds or captures mineral N originating from organic matter would have accounted for increased leaching [38]. In Sweden and Denmark, delaying the incorporation of ryegrass as catch crop effectively reduced N leaching [19, 39].

### Effects of weather

Of the three winters, that of 2011–2012 was the highest wettest, warmest, most subject to freeze-thaw cycles, and produced the lowest N.BS. Rainfall is well known to increase N leaching, as already discussed, and increased activity of microorganisms due to the higher temperatures could have increased immobilization [40]. The effect of freeze-thaw cycles could be investigated further. Use of winter cover crops, such as winter turnip rape, can reduce N leaching [41].

## Conclusion

When only two-thirds of the recommended rate of N fertilization was used on barley, the break-crop effects of turnip rape, faba bean, white lupin and hemp were shown in enhanced yield and/or protein concentration. Some combinations of two years of break crops had greater effects than a single year. This confirms the importance of break crops in cereal-based agriculture, even at high latitudes where many disease and pest pressures are considered to be low. As shown by the low covariance, little of the effect of the break crop was attributable to available N in plough layer at sowing. It would be interesting to investigate dynamics of other elements after rotation with faba bean, white lupin and hemp. Mineral N derived from the soil organic matter and plant residues was at risk of loss in mild, wet winters and springs. Incorporation of green manure resulted in a slight increase of barley grain protein concentration, but posed a great potential for increasing mineral N loss and did not result in higher yield of following barley compared with incorporation of residues after harvest. Hence, we recommend either incorporation of harvest residues rather than green manure, or using a catch crop such as winter turnip rape to reduce nutrient leaching.

## Supporting Information

S1 Table Mineral nitrogen concentration two months after incorporation of plant materials (N.2), before sowing the barley crop (N.BS), and difference (N.D) between N.2 and N.BS at two sites and after three first crops. Data show means across replicates, second crops and incorporation times.
(DOC)

S2 Table Mineral nitrogen concentration two months after incorporation of plant materials (N.2), before sowing the barley crop (N.BS), and difference (N.D) between N.2 and N.BS at two sites and after six second crops. Data show means across replicates, sites and incorporation times.
(DOC)

S3 Table Yield and grain protein content (GPC) of barley after three first crops and six second crops. Data show means across replicates, sites and times of second crop incorporation.
(DOC)

S4 Table Mineral nitrogen concentration two months after incorporation of plant materials (N.2), and difference (N.D) between N.2 and N.BS at two sites and after two times of incorporating residues of second crops. Data show means across replicates, first crops and second crops
(DOC)

S5 Table Mean squares and significance levels of terms in ANOVA of yield and grain protein concentration (GPC) of barley and mineral nitrogen concentration two month after incorporation of plant materials (N.2), before sowing the barley crop (N.BS), and difference (N.D) between N.2 and N.BS. (DOCX)

S6 Table Yields and grain protein concentration of third-year barley, mineral N two months after incorporation of crops (N.2), mineral N before sowing third-year barley (N.BS) and mineral N difference between N.2 and N.BS (n = 4). (XLSX)

## References

[1] Dalal RC, Mayer RJ. Long-term trends in fertility of soils under continuous cultivation and cereal cropping in southern Queensland. VI. Loss of total nitrogen from different particle-size and density fractions. Aust. J. Crop. Sci. 1987; 25: 83–93.

[2] Johnston AE, Poulton PR, Coleman K. Soil organic matter: its importance in sustainable agriculture and carbon dioxide fluxes. Adv. Agron. 2009; 101: 1–57.

[3] Tekauz A. Diseases of barley In: Diseases of field crops in Canada. Canadian Phytopathological Society and University Extension Press, University of Saskatchewan, Saskatoon, Sask., Canada 2003; pp. 30–53.

[4] Ministry of Agriculture and Forestry of Finland. 25 Nov 2010. Cereal production. Available:http://www.mmm.fi/en/index/frontpage/Agriculture/agricultural_production/plant_production. Accessed 12 June 2014.

[5] Kirkegaard J, Christen O, Krupinsky J, Layzell D. Break crop benefits in temperate wheat production. Review. Field Crops Res. 2008; 107:185–195.

[6] Peoples MB, Brockwell J, Herridge DF, Rochester IJ, Alves BJR, Urquiaga S, et al The contributions of nitrogen-fixing legumes to the productivity of agricultural systems. Symbiosis. 2009; 48:1–17.

[7] Wink M. Inhibition of seed germination by quinolizidine alkaloids—Aspects of allelopathy in *Lupinus albus* L. Planta.1983; 158: 365–368. 10.1007/BF00397339 24264757

[8] Azirak S. Karaman S. Allelopathic effect of some essential oils and components on germination of weed species. Acta Agric. Scand. Sect. B Soil Plant Sci. 2008; 58: 88–92.

[9] Inam B, Hussain F, Farhat B. *Cannabis sativa* L. is allelopathic. Pakistan J. Sci. Industrial Res. 1989; 32: 617–620.

[10] Campiglia E, Mancinelli R, Di Felice V, Radicetti E. Long-term residual effects of the management of cover crop biomass on soil nitrogen and yield of endive (*Cichorium endivia* L) and savoy cabbage (*Brassica oleracea* var. *sabauda*). Soil Tillage Res. 2014; 139: 1–7.

[11] Macías FA, Molinillo JMG, Varela RM, Galindo JCG. Allelopathy—A natural alternative for weed control. Pest Manag. Sci. 2007; 63: 327–348. 17348068 10.1002/ps.1342

[12] Hamza MA, Anderson WK. Soil compaction in cropping systems: A review of the nature, causes and possible solutions. Soil Tillage Res. 2007; 82:121–45.

[13] Lovett JV. 1991 Changing perceptions of allelopathy and biological control. Biol. Agric. Hortic. 8: 89–100.

[14] Chen Y, Zhu Z, Guo Q, Zhang L, Zhang X. 2012 Variation in concentrations of major bioactive compounds in *prunella vulgaris* L. related to plant parts and phenological stages. Biol Res. 45: 171–5. 10.4067/S0716-97602012000200009 23096361

[15] FAO. World Reference Base for Soil Resources 2006, first update 2007. World Soil Resources Reports No. 103. FAO, Rome

[16] Meier U. 2001. Growth stages of mono-and dicotyledonous plants BBCH Monograph. Available:http://wwwjkibundde/fileadmin/dam_uploads/_veroeff/bbch/BBCH-Skala_englischpdf

[17] R Development Core Team. 2009 R: A language and environment for statistical computing. R Foundation for Statistical Computing, Vienna, Austria ISBN 3-900051-07-0, Available: http://wwwR-projectorg.

[18] Felipe M. 2012. Statistical Procedures for Agricultural Research. Available: http://cranr-projectorg/web/packages/agricolae/agricolaepdf.

[19] Stenberg M, Aronsson H, Lindén B, Rydberg T, Gustafson A. Soil mineral nitrogen and nitrate leaching losses in soil tillage systems combined with a catch crop. Soil Tillage Res. 1999; 50: 115–125.

[20] Paasonen-Kivekäs M, Yli-Halla M. A comparison of nitrogen and carbon reserves in acid sulphate and non-acid sulphate soils in western Finland. Agric. Food Sci. Finland. 2005; 14: 57–69.

[21] Salo T, Turtola E. Nitrogen balance as an indicator of nitrogen leaching in Finland. Agric. Ecosyst. Environ. 2006; 113: 98–107.

[22] Kraljević D, Šumanovac L, Heffer G., Horvat Z. Effect of precrop on winter wheat yield. Cereal Res. Commun. 2007; 35: 665–668.

[23] Kaul HP. Pre-crop effects of grain legumes and linseed on soil mineral N and productivity of subsequent winter rape and winter wheat crops. Bodenkultur. 2004; 55: 95–102.

[24] Zou L, Santanen A, Tein B, Stoddard FL, Mäkelä PSA. Interference potential of buckwheat, fababean, oilseed hemp, vetch, white lupine and caraway to control couch grass weed. Allelopathy J. 2014; 33: 227–236.

[25] Mandal N, Dwivedi BS, Meena MC, Singh D, Datta SP, Tomar RK, et al Effect of induced defoliation in pigeonpea, farmyard manure and sulphitation pressmud on soil organic carbon fractions, mineral nitrogen and crop yields in a pigeonpea-wheat cropping system. Field Crops Res. 2013; 154: 178–187. 10.1007/s12011-013-9724-6 23771645

[26] Miransari M, Mackenzie AF. Corn (*Zea mays* L.) grain and stover yield as affected by soil residual mineral nitrogen. Commun. Soil Sci. Plant Anal. 2012; 43: 799–810.

[27] St Luce M, Ziadi N, Zebarth BJ, Whalen JK, Grant CA, Gregorich EG, et al Particulate organic matter and soil mineral nitrogen concentrations are good predictors of the soil nitrogen supply to canola following legume and non-legume crops in western Canada. Can. J. Soil Sci. 2013; 93: 607–620.

[28] Hauggaard-Nielsen H, Mundus S, Jensen ES. Nitrogen dynamics following grain legumes and subsequent catch crops and the effects on succeeding cereal crops. Nutri. Cycl. Agroecosyst. 2009; 84: 281–291.

[29] Kramberger B, Gselman A, Janzekovic M, Kaligaric M, Bracko B. Effects of cover crops on soil mineral nitrogen and on the yield and nitrogen content of maize. Eur. J. Agron. 2009; 31: 103–109.

[30] Arcand MM, Knight JD, Farrell RE. Estimating belowground nitrogen inputs of pea and canola and their contribution to soil inorganic N pools using ^15^N labeling. Plant Soil. 2013; 371: 67–80.

[31] Valkama E., Salo T., Esala M., Turtola E. Nitrogen balances and yields of spring cereals as affected by nitrogen fertilization in northern conditions: A meta-analysis. Agric. Ecosyst. Environ. 2013; 164: 1–13.

[32] Neumann G, Massonneau A, Langlade N, Dinkelaker B, Hengeler C, Römheld V, et al Physiological aspects of cluster root function and development in phosphorus-deficient white lupin (*Lupinus albus* L). Ann. Bot. 2000; 85: 909–919.

[33] Hinsinger P. Bioavailability of soil inorganic P in the rhizosphere as affected by root-induced chemical changes: A review. Plant Soil. 2001; 237: 173–195.

[34] Heikinkeimo M., Fougstedt B. Statistic of soil temperature in Finland 1971–1990 Finnish Meteorological Institute. Meteorological Publications 1992; 22: 75.

[35] Pietikainen J., Pettersson M., Baath E. Comparison of temperature effects on soil respiration and bacterial and fungal growth rates. FEMS Microbio. Ecol. 2005; 52: 49–58. 16329892 10.1016/j.femsec.2004.10.002

[36] Turtola E., Kemppainen E. Nitrogen and phosphorus losses in surface runoff and drainage water after application of slurry and mineral fertilizer to perennial grass ley. Agric. Food Sci. Finland. 1998; 7: 569–581.

[37] Lindén B., Lyngstad I., Sippola J., Søegaard K., Kjellerup V. Nitrogen mineralisation during the growing season. I. Contribution to the nitrogen supply of spring barley. Swedish J. Agric Res. 1992; 22: 3–12.

[38] Yläranta T, Uusi-Kämppä J, Jaakkola A. Leaching of nitrogen in barley, grass ley and fallow lysimeters. Agric. Food Sci. Finland. 1993; 2: 281–291.

[39] Thorup-Kristensen K, Dresbøll DB. Incorporation time of nitrogen catch crops influences the N effect for the succeeding crop. Soil Use Manag. 2010; 26: 27–35.

[40] Pietikainen J., Pettersson M., Baath E. Comparison of temperature effects on soil respiration and bacterial and fungal growth rates. FEMS Microbiol Ecol. 2005; 52: 49–58. 16329892 10.1016/j.femsec.2004.10.002

[41] Weinert TL, Pan WL, Moneymaker MR, Santo GS, Stevens RG. Nitrogen recycling by non-leguminous winter cover crops to reduce leaching in potato rotations. Agron. J. 2002; 94: 365–372.

