# Supplementary material for: Effects of Break Crops on Yield and Grain Protein Concentration of Barley in a Boreal Climate
Source: PLoS One. 2015 Jun 15;10(6):e0130765. doi: 10.1371/journal.pone.0130765 (PMC4468161; doi:10.1371/journal.pone.0130765)
Supplement: S1 Table — Data show means across replicates, second crops and incorporation times. (DOC) [file pone.0130765.s001.doc]

**S1 Table. Mineral nitrogen concentration two months after incorporation of plant materials (N.2), before sowing the barley crop (N.BS), and difference (N.D) between N.2 and N.BS at two sites and after three first crops.** Data show means across replicates, second crops and incorporation times.

| Site | First crop | N.2 (kg/ha) |  | N.BS (kg/ha) |  | N.D (kg/ha) |  |
| --- | --- | --- | --- | --- | --- | --- | --- |
| I | Barley | 35.2 | b | 19.9 | d | 15.2 | b |
|  | Turnip rape | 53.5 | a | 26.3 | bc | 26.8 | a |
|  | Faba bean | 52.0 | a | 23.9 | c | 28.1 | a |
| II | Barley | 36.5 | b | 28.7 | ab | 6.9 | bc |
|  | Turnip rape | 34.4 | b | 29.1 | ab | 3.6 | c |
|  | Faba bean | 35.7 | b | 29.9 | a | 3.3 | c |
| Within a column, means followed by the same letter are not significantly different (P < 0.05) by the LSD test. | | | | | | | |
