# Supplementary material for: Effects of Break Crops on Yield and Grain Protein Concentration of Barley in a Boreal Climate
Source: PLoS One. 2015 Jun 15;10(6):e0130765. doi: 10.1371/journal.pone.0130765 (PMC4468161; doi:10.1371/journal.pone.0130765)
Supplement: S3 Table — Data show means across replicates, sites and times of second crop incorporation. (DOC) [file pone.0130765.s003.doc]

**S3 Table. Yield and grain protein content (GPC) of barley after three first crops and six second crops.** Data show means across replicates, sites and times of second crop incorporation.

| First crop | Second crop | Yield (kg/ha) | | GPC (%) | |
| --- | --- | --- | --- | --- | --- |
| Barley | Barley | 4799 | bc | 9.1 | hij |
|  | Buckwheat | 4752 | c | 8.8 | j |
|  | Caraway | 4696 | c | 9.1 | ij |
|  | Faba bean | 5157 | abc | 9.2 | ghi |
|  | Hemp | 4838 | bc | 8.8 | j |
|  | White lupin | 5166 | abc | 8.8 | j |
| Turnip rape | Barley | 4884 | abc | 9.9 | bcd |
|  | Buckwheat | 5200 | abc | 10.0 | abcd |
|  | Caraway | 5317 | ab | 10.1 | abc |
|  | Faba bean | 5339 | a | 10.2 | ab |
|  | Hemp | 5290 | ab | 9.8 | cde |
|  | White lupin | 4964 | abc | 10.3 | a |
| Faba bean | Barley | 5060 | abc | 9.3 | ghi |
|  | Buckwheat | 5046 | abc | 9.5 | efg |
|  | Caraway | 5332 | ab | 9.3 | fghi |
|  | Faba bean | 5272 | ab | 10.0 | abc |
|  | Hemp | 5442 | a | 9.4 | fgh |
|  | White lupin | 5407 | a | 9.7 | def |

Within a column, means followed by the same letter are not significantly different (P < 0.05) by the LSD test.
