# Supplementary material for: Effects of Break Crops on Yield and Grain Protein Concentration of Barley in a Boreal Climate
Source: PLoS One. 2015 Jun 15;10(6):e0130765. doi: 10.1371/journal.pone.0130765 (PMC4468161; doi:10.1371/journal.pone.0130765)
Supplement: S4 Table — Data show means across replicates, first crops and second crops (DOC) [file pone.0130765.s004.doc]

**S4 Table. Mineral nitrogen concentration two months after incorporation of plant materials (N.2), and difference (N.D) between N.2 and N.BS at two sites and after two times of incorporating residues of second crops.** Data show means across replicates, first crops and second crops

| Site | Stage of incorporation | N.2 (kg/ha) |  | N.D (kg/ha) |  |
| --- | --- | --- | --- | --- | --- |
| I | Flowering | 55.5 | a | 31.6 | a |
| I | After harvest | 36.1 | b | 13.0 | c |
| II | Flowering | 51.3 | a | 19.8 | b |
| II | After harvest | 15.8 | c | -14.4 | d |

Within a column, means followed by the same letter are not significantly different (P < 0.05) by the LSD test.
