# Supplementary material for: Effects of Break Crops on Yield and Grain Protein Concentration of Barley in a Boreal Climate
Source: PLoS One. 2015 Jun 15;10(6):e0130765. doi: 10.1371/journal.pone.0130765 (PMC4468161; doi:10.1371/journal.pone.0130765)
Supplement: S5 Table — (DOCX) [file pone.0130765.s005.docx]

| Sources of variation | Df | MS (Yield) |  | MS (GPC) |  | MS (N.2) |  | MS (N.BS) |  | MS (N.D) |  |
| --- | --- | --- | --- | --- | --- | --- | --- | --- | --- | --- | --- |
| Replicate | 3 | 0.14 |  | 0.09 |  | 151 |  | 53 |  | 398 |  |
| Site | 1 | 55.34 | *** | 0.37 |  | 6984 | *** | 1826 | *** | 19044 | *** |
| First crop | 2 | 2.57 | *** | 21.40 | *** | 1560 | *** | 225 | *** | 472 | * |
| Second crop | 5 | 0.58 | ** | 1.55 | *** | 3744 | *** | 166 | *** | 1897 | *** |
| Stage of incorporation | 1 | 0.06 |  | 2.33 | *** | 32077 | *** | 139 | * | 37050 | *** |
| Site × First crop | 2 | 0.07 |  | 0.07 |  | 2211 | *** | 163 | ** | 1468 | *** |
| Site × Second crop | 5 | 0.01 |  | 0.05 |  | 1091 | *** | 123 | *** | 251 |  |
| First crop × Second crop | 10 | 0.31 | * | 0.32 | * | 100 |  | 19 |  | 110 |  |
| Site × Stage of incorporation | 1 | 0.00 |  | 0.00 |  | 2228 | *** | 389 | *** | 4763 | *** |
| First crop × Stage of incorporation | 2 | 0.13 |  | 0.10 |  | 654 | * | 42 |  | 497 | * |
| Second crop × Stage of incorporation | 2 | 0.25 |  | 1.70 | *** | 873 | ** | 72 |  | 424 |  |
| Site × First crop × Second crop | 10 | 0.00 |  | 0.04 |  | 113 |  | 64 | ** | 193 |  |
| Site × First crop × Stage of incorporation | 2 | 0.00 |  | 0.18 |  | 539 | * | 35 |  | 947 | ** |
| Site × Second crop × Stage of incorporation | 2 | 0.00 |  | 0.19 |  | 100 |  | 46 |  | 235 |  |
| First crop × Second crop × Stage of incorporation | 4 | 0.47 | * | 0.05 |  | 103 |  | 45 |  | 112 |  |
| Site × First crop × Second crop × Stage of incorporation | 4 | 0.00 |  | 0.11 |  | 30 |  | 73 | * | 132 |  |
| Residuals | 159 | 0.16 |  | 0.14 |  | 149 |  | 25 |  | 152 |  |

**S5 Table. Mean squares and significance levels of terms in ANOVA of yield and grain protein concentration (GPC) of barley and mineral nitrogen concentration two month after incorporation of plant materials (N.2), before sowing the barley crop (N.BS), and difference (N.D) between N.2 and N.BS.**

*, **, ***: P < 0.05, 0.01, 0.001, respectively.
